# Differentiation between genetic mutations of breast cancer by breath volatolomics

**DOI:** 10.18632/oncotarget.6269

**Published:** 2015-11-02

**Authors:** Orna Barash, Wei Zhang, Jeffrey M. Halpern, Qing-Ling Hua, Yue-Yin Pan, Haneen Kayal, Kayan Khoury, Hu Liu, Michael P.A. Davies, Hossam Haick

**Affiliations:** ^1^ Department of Chemical Engineering and Russel Berrie Nanotechnology Institute, Technion-Israel Institute of Technology, Haifa, Israel; ^2^ Department of Oncology, the First Affiliated Hospital of Anhui Medical University, Anhui, China; ^3^ Molecular & Clinical Cancer Medicine, University of Liverpool, Cancer Research Centre, Liverpool, United Kingdom

**Keywords:** breast cancer, molecular, volatolomic, sensor, spectrometry

## Abstract

Mapping molecular sub-types in breast cancer (BC) tumours is a rapidly evolving area due to growing interest in, for example, targeted therapy and screening high-risk populations for early diagnosis. We report a new concept for profiling BC molecular sub-types based on volatile organic compounds (VOCs). For this purpose, breath samples were collected from 276 female volunteers, including healthy, benign conditions, ductal carcinoma *in situ* (DCIS) and malignant lesions. Breath samples were analysed by gas chromatography mass spectrometry (GC-MS) and artificially intelligent nanoarray technology. Applying the non-parametric Wilcoxon/Kruskal-Wallis test, GC-MS analysis found 23 compounds that were significantly different (*p* < 0.05) in breath samples of BC patients with different molecular sub-types. Discriminant function analysis (DFA) of the nanoarray identified unique volatolomic signatures between cancer and non-cancer cases (83% accuracy in blind testing), and for the different molecular sub-types with accuracies ranging from 82 to 87%, sensitivities of 81 to 88% and specificities of 76 to 96% in leave-one-out cross-validation. These results demonstrate the presence of detectable breath VOC patterns for accurately profiling molecular sub-types in BC, either through specific compound identification by GC-MS or by volatolomic signatures obtained through statistical analysis of the artificially intelligent nanoarray responses.

## INTRODUCTION

Breast cancer (BC) is promoted by the interplay of hereditary and environmental risk factors that cause progressive accumulation of genetic and epigenetic changes in breast cells, *e.g.* inactivating DNA repair genes [1]. BC-promoting DNA methylation of certain genes can have occurred in pre-malignant lesions, indicating that epigenetic changes arise very early in BC tumorigenesis [1, 2]. Hence, BC and BC precursors may be associated with their own characteristic signature of gene expression.

These gene signatures include the hormone receptors [estrogen receptor (ER) or progesterone receptor (PgR)] and epidermal growth factor receptors [ErbB-1 (EGFR) and ErbB-2 (ERBB2, HER-2/neu, HER2)] as predictive biomarkers related to targeted therapy of BC [1, 3–5]. Gene expression cDNA microarray studies indicated that within the hormone-receptor positive (luminal) tumours there are at least 2 subtypes, Luminal A and B. Each subtype is characterized with a distinct gene expression and prognosis, Luminal B giving poorer outcomes [6, 7]. Immunohistochemical markers can be used as surrogates for the microarray-based profiles, including the proliferation marker, Ki-67, higher levels defining the Luminal B subtype (which also tends to be HER2 positive) [7]. In human BC, EGFR overexpression is commonly linked to HER2 overexpression [4], HER2 positivity being a predictive marker for treatment with specific HER2-targeted therapies, e.g. trastuzumab [8]. Some BC tumours may have HER2 overexpression and/or amplified DNA, and these fall into the Luminal B (for hormone-receptor positive) or HER2+ subtype (if non-luminal) [7]. Approximately 75–80% of BC tumours are positive for ER and/or PgR, whereas 20–25% of the tumours are receptor-negative or triple-negative when not overexpressing ER, PgR and HER2. The molecular subtypes defined by positive hormone receptor status are used to guide anti-estrogen therapies, such as tamoxifen and anastrazole [9]. The present classification of breast cancer into subgroups relies mainly on gene expression patterns that require a suitable amount and quality of tumour tissue. However, the widespread use of gene expression profiling for research and clinical diagnosis of molecular sub-types remains limited due to high cost and technical difficulties. The expression level of many genes is subject to post-transcriptional upregulation or downregulation, i.e. gene sequencing does not always correspond with protein levels [10]. Moreover, while it is possible to yield direct biochemical insight by studying the proteins, there remain some unavoidable problems such as limited and variable sample material, sample degradation, a plethora of issues arising from post-translational modifications, large dynamic range, and disease and drug perturbations [11]. Further development of mass spectrometric technology is needed that increases sensitivity, robustness and data handling.

A promising approach for achieving accurate, time-efficient and inexpensive molecular sub-type classification of BC tumours relies on volatolomic studies by analysing volatile organic compounds (VOCs) [12–18]. The rationale behind this approach is that cancer-associated gene and protein changes can lead to oxidative stress [14, 18], with different cell membranes causing disease-specific VOCs exuded into the blood [12–14, 18]. Some of these VOCs mixed in the blood are of distinctively different compositions, depending on whether a cell is healthy or cancerous, and they will be exhaled in the breath [12–27].

The ability of breath volatolomics to discriminate between malignant tumours and non-malignant tumour of BC patients has been further assessed [25, 28–31]. It gives a means of discriminating between different molecular sub-types of BC that would be particularly suitable for patient risk assessment, as well as for defining a suitably targeted therapy for BC patients, particularly in advanced disease where biopsy of metastatic tumours is difficult. These considerations will also be assessed here [25, 28–31], including an assessment of the detection and differentiation between Luminal A, Luminal B, Triple Negative and HER2+ molecular sub-types, and a small number defined as non-luminal, HER2 equivocal, having positive immunohistochemistry (IHC) without confirmatory Fluorescence In-Situ Hybridization (FISH) analysis.

## RESULTS

**Figure 1:**
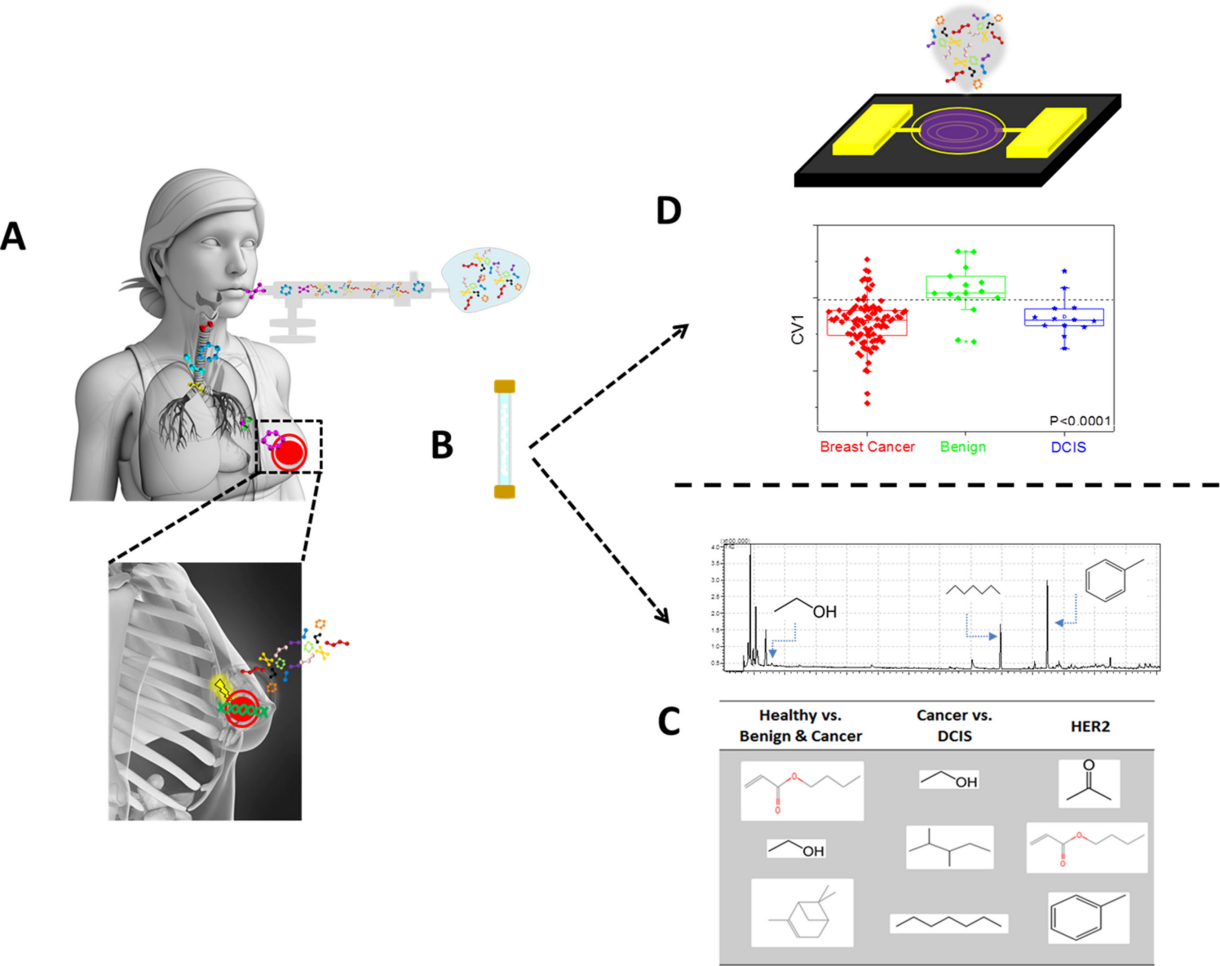
Schematic figure describing the collection and analysis procedures of the exhaled breath using two approaches Following lung-wash the patient inhale into a collection bag (**A**), which is then being collected and concentrated on Tenax^®^ TA sorption tubes (**B**). The sorbent tube is then exposed both to GC-MS for specific compound identification (**C**) and to artificially intelligence nanoarray for volatolomic signature of breast cancer genetic mutations (**D**).

**Figure 2:**
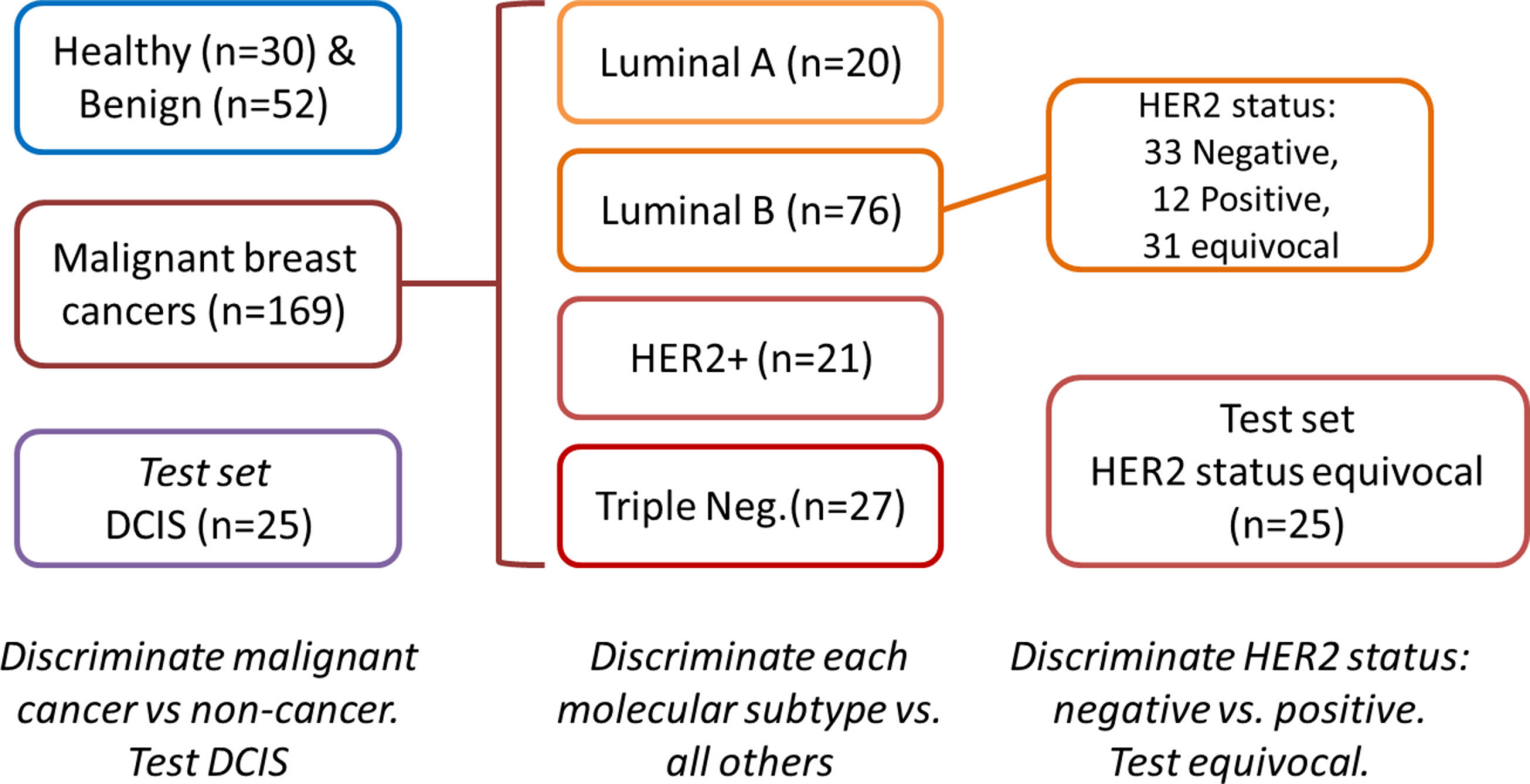
Study design

Breath samples were collected and monitored in a reproducible manner, as established from a study monitoring breath samples variance from day-to-day for over 6 months. Gas chromatography mass spectrometry (GC-MS) analysis detected small variation (± 4%) in breath composition, indicating the high accuracy and reproducibility of breath collection, notably being unaffected by differences in place and time. The effect of room air on breath samples also proved negligible. (Further information can be found in the text and supporting information in [19]). The collected breath samples from different molecular sub-types BC patients were analysed by 2 different and independent methods (Table 1, Figures 1 & 2). The first method is based on chemical analysis by GC-MS for the identification and quantification of the variety of VOCs in exhaled breath. The second method is based on cross-reactive nanoarrays in combination with pattern recognition (so called artificially intelligent nanoarrays). This approach provides collective VOC patterns as opposed to identification and quantification of specific VOCs.

**Table 1:** Clinical characteristics of enrolled subjects

Category		Characteristics	Age (Median, Min-Max Range)	No. in GC-MS	No. in artificially intelligent nanoarray- Group A	No. in artificially intelligent nanoarray- Group B
Non-Malignant	Healthy	No breast disease	42, 26–74	23	−	30
	Benign	non-cancerous lesions	42, 24–62	13	37	15
	DCIS	ductal carcinoma *in situ*	46, 32–70	10	12	13
Malignant	LuminalA	ER+ *; PgR +^†^; Ki–67^‡^ ≤ 14%	48, 34–69	11	8	12
	LuminalB	ER+ ; PgR+ ; Ki–67 > 14% or HER2 ** ++ or +++	48, 25–69	34	34	42
	Triple Neg.	ER− ; PgR− ; HER2− or −/+ IHC or HER2++ IHC^^^ & FISH ^^^^ negative	49, 21–69	10	15	12
	HER2+^‡^	ER− ; PgR− ; HER2+++ IHC or HER2++ IHC & FISH positive	50, 32–67	15	5	16
	HER2 equivocal	ER− ; PgR− ; HER2++ IHC & FISH not available	51, 34–63	10	11	14

Healthy volunteers, patients with benign lesions (which include adenosis, apocrine metaplasia, ductal hyperplasia, fibroadenoma, granulomatous inflammation intraductal papilloma, lobular hyperplasia, and mastitis), ductal carcinoma *in situ* (DCIS) and BC patients classified into different molecular sub-types (Luminal A, Luminal B, Triple Negative, HER2+ and HER2 equivocal).

* ER = Estrogen Receptor

† PgR = Progesterone Receptor

‡ Ki-67 = proliferation marker

** HER2 = Human epidermal growth factor receptor 2

^ IHC = Immunohistochemistry

^^ FISH = Fluorescence In-Situ Hybridization

### GC-MS analysis

Analysis of breath VOCs by GC-MS tentatively identified > 500 different VOCs in each breath sample. Analysis using a GC-MS post-run program identified 132 VOCs regularly found in > 80% of the samples. Non-parametric Wilcoxon/Kruskal-Wallis tests identified 23 VOCs that were significantly different between room and breath samples, and provided the database for multiple binary comparisons based on area-under-curve quantitation values obtained by GC-MS (Table S1 & S2, SI). Among these VOCs, 21 were significantly different in healthy volunteers compared to patients with breast lesions (benign conditions, DCIS and BC). An overlapping set of 14 VOCs were significantly different between BC and non-BC patients (i.e. those with malignant disease rather than benign disease or no disease), indicating most of these VOCs were cancer specific. In contrast, there were very few differences between DCIS and malignant BC (only 4 VOCs), in keeping with the close relationship between these types of tumour (Table S1). No compound was significantly different in a comparison of Luminal A or B with other cancers, or between patients with either Luminal A or Luminal B cancers. However, a number of VOC were associated with Triple Negative and HER2 status (Table S1). Of note, those related to HER2 status in luminal and non-luminal BC were different. Quantitative values of compounds detected by GC-MS were determined by calibration curves of external standards (see SI, section S1 & Table S2 for more information).

Further analysis of the raw data show that combining multiple GC-MS signals by DFA can more powerfully differentiate patients with different types of breast lesions [32, 33]. With DFA analysis and leave-one-out cross-validation on the set of 23 compounds, we identified 14 compounds that provided discrimination of 78% sensitivity and 72% accuracy compared with healthy volunteers and patients with benign conditions relative to a group of cancer patients (Table 2). Combining BC patients with patients having benign lesions and then comparing them to healthy controls, sensitivity and accuracy dramatically improve to 86 and 83%, respectively (Table 2). Consistent with the single compound analysis, combinations of VOCs related to HER2 status in luminal and non-luminal BC were different. Higher sensitivities were observed for the HER2 status in luminal and non-luminal BC independently compared to HER2 status across both groups. DFA combination of only 2 compounds (acetone and 2, 3-dimethyl-pentane), resulted in maximum sensitivity (100%) for identifying breast cancer patients having non-luminal HER2+ status. Three different VOCs (carbonic acid, dimethyl ester and 1, 4-dimethyl-cyclohexane) used in identifying BC patients having luminal HER2+ gave a sensitivity of 73% (Table 2). Together with the different VOCs found in luminal and non-luminal BC, this indicates subtle differences in the impact of HER2 on different molecular subtypes. A high specificity (95%) was observed in comparing triple negative versus other cancers. However, there was a low sensitivity (40%), probably due to the small number of positive results (only 10 cases of triple negative, compared to 60 “other” cases). The same reason might also account for the relatively low sensitivity of HER2+ compared to other cancers. Calculated *P*-values of the Wilcoxon test for the 1st canonical variable (CV1) were considerably lower than 0.05 for all binary comparisons with Receiver Operating Characteristic - Area Under Curve (ROC-AUC) values of 0.82–0.93 (Table 2).

**Table 2:** List of 14 VOCs used to for multiple binary comparisons

Suspected VOC	Healthy *vs.* Benign + Cancer	Healthy + Benign *vs.* Cancer	Cancer *vs.* DCIS	Triple Negative *vs.* Other	HER2+ *vs.* Other	HER2 + status* *vs.* Other	HER2+ status (Non Luminal) *vs.* Other	HER2+ status (Luminal) *vs.* Other
Ethanol	**X**	**X**	**X**			**X**		
Acetone					**X**		**X**	
Cyclopentane				**X**				
Carbonic acid, dimethyl ester (DMC)					**X**	**X**		**X**
Pentane, 2, 3-dimethyl-			**X**				**X**	
Heptane		**X**	**X**					
Toluene						**X**		
Cyclohexane, 1, 4-dimethyl-				**X**				**X**
Acetic acid, butyl ester				**X**				
Benzene, 1, 3-dimethyl-					**X**	**X**		
2-Propenoic acid, butyl ester	**X**	**X**						
alpha.-Pinene	**X**				**X**			
5-Hepten-2-one, 6-methyl-		**X**						
1-Hexanol, 2-ethyl-			**X**					
**Sensitivity**	**86**	**78**	**70**	**40**	**67**	**68**	**100**	**73**
**Specificity**	**70**	**61**	**83**	**95**	**76**	**72**	**57**	**70**
**Accuracy**	**83**	**72**	**81**	**87**	**74**	**70**	**82**	**72**
***p*-value**	**< 0.0001**	**< 0.0001**	**0.0001**	**0.006**	**0.0001**	**< 0.0001**	**0.0047**	**0.0018**
**AUC**	**0.93**	**0.79**	**0.88**	**0.78**	**0.83**	**0.82**	**0.91**	**0.87**

The groups have previously been described. Classification success calculated for the CV values obtained from DFA analysis of the GC-MS values expressed in range of values for two independent studies. Sensitivity, specificity, accuracy *p*-value and AUC were calculated according to the confusion matrix of each study separately.

* HER2+ status related VOC identified based on HER2 negative (IHC− or −/+) vs. HER2+ (IHC+++ or IHC++/FISH+).

### Analysis with artificially intelligent nanoarray

**Figure 3:**
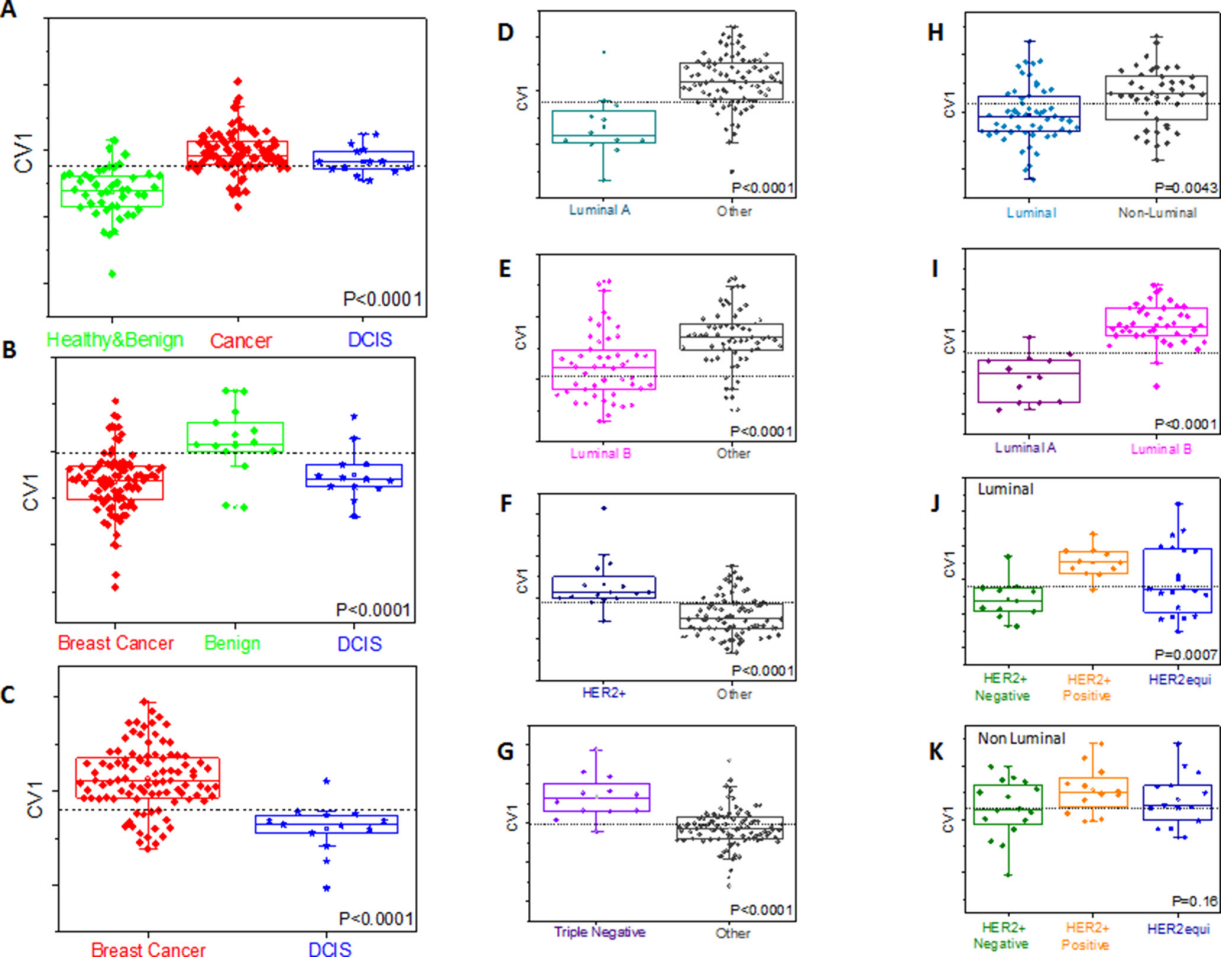
Representative DFA plots of CV values obtained from the response of the sensor array to breath VOCs from different sub-groups (Group B) The boxes represent 95% CI of CV values; error bars represent the standard deviation. Central dotted line represents Youden's cut-point. Each graph refers to different comparison. Values are given in Table 3 and Table S3 of SI. Comparisons are shown for breast cancer with healthy and benign (**A**) benign (**B**) and DCIS (**C**) cases [DCIS have been treated as blind samples in A and B]. Comparisons are also shown for each molecular sub-group of breast cancer with all other types (**D–G**) distinction between luminal and non-luminal cancers (**H**) and between different luminal types (**I**) distinction of HER2 status within luminal (**J**) and non-luminal (**K**) breast cancers.

Volatolomic signatures of breath VOCs based on genetic expression subtypes were determined using artificially intelligent nanoarray based on Gold Nanoparticles (GNPs) and Single Wall Carbon Nanotubes (SWCNTs) coated with different organic layers. The collective response from the array was analysed using DFA to obtain unique volatolomic signatures of each BC sub-type. DFA could discriminate between every 2 previously defined sets of samples (Figure 3). Breath samples were collected in 2 batches that were stored under different conditions. To avoid influence of environmental confounding factors, breath samples were divided into 2 groups (A & B) for further analysis (SI, Table S3) and mean accuracy, sensitivity and specificity of the range of values are given in Table 3.

**Table 3:** Classification success calculated for the CV values obtained from DFA analysis of the sensor array responses from 2 independent studies, expressed as range of values

Comparison	Training/Test	Sample size	Accuracy [%]	Sensitivity [%]	Specificity [%]	AUC	Test group
Healthy + Benign *vs.* Cancer	Training	140	88.3	90.6	83.3	0.91	11 of 13 (85%) DCIS as cancer
	Blind Test		83	84	80	0.90	
Benign *vs.* Cancer	Training	221	71.2–82	62.2–80	75.7–82.3	0.73–0.82	17 of 25 (68%) DCIS as cancer
Cancer *vs.* DCIS	Training	194	81.4–84.4	83–83.3	81.1–92	0.81–0.89	-
Luminal A *vs.* Other	Training	169	81.3–87.7	75–87.5	82.1–87.5	0.83–0.87	-
Luminal B *vs.* Other	Training	169	78.1–86.3	83.3–85.3	74.1–87.2	0.83–0.84	-
Triple Negative *vs.* Other	Training	144	82.9–90.3	83.3–93.3	82.9–89.4	0.87–0.91	-
HER2+ *vs.* Other	Training	164	81.3–82.4	81.3–91	80.7–81.3	0.85–0.86	-
HER2+ status*	Training	110	80.7–95.8	77.8–100	82.9–95.1	0.85–0.99	12 of 34 (35%) HER2 equivocal as HER2+
HER2+ status (Non-Luminal)	Training	22	90.9	90.9	90.9	0.93	8 of 20 (40%) HER2 equivocal as HER2+
HER2+ status (Luminal)	Training	48	85.7–100	85.7–100	83.3–100	0.66–1	8 of 25 (32%) HER2 equivocal as HER2+
Luminal *vs.* Non-Luminal	Training	169	70.8–87.7	70.4–88.1	71.4–87.1	0.67–0.86	-
Luminal A *vs.* Luminal B	Training	98	85.7–94	75–91.7	88.2–95.2	0.87–0.96	-

Sensitivity, specificity, accuracy and AUC were calculated separately according to the confusion matrix of each study (further information in SI, Table S2). Ranges are given here.

* HER2+ status related VOC identified based on HER2 negative (IHC− or −/+) *vs.* HER2+ (IHC+++ or IHC++/FISH)

It was possible to discriminate BC from benign breast disease (accuracy 71%–82%) using leave-one-out cross-validation on a training set, with higher accuracy (88%) when healthy volunteers were included. Results of a blind test of one-third of the samples yielded 83% accuracy, 84% sensitivity and 80% specificity. ROC analysis of canonical variable 1 (CV1) of the blind test gave ROC-AUC of 0.90 and the calculated AUC was 0.91 for the training set. CV1 values were significantly different between these 2 tested groups, both for the training and the tests with Wilcoxon *P*-value of < 0.0001 (Table 3; and SI, Table S3). DCIS samples defining pre-malignant lesions (for which the treatment decisions are different) were not included within the malignant disease. Indeed, DCIS states are closely related to invasive breast cancer; including them with the benign diseases would limit our ability to discriminate. We, therefore, treated DCIS as an independent test set, excluding them from cases used to define the disease/normal signature, and investigating how that signature defines them. In both cases when DCIS samples were tested, the majority were identified as cancer on the basis of their DFA CV1 values, underlining the similarity between DCIS and malignant disease. It was possible to construct an accurate DFA that discriminates between DCIS lesions and cancer (Table 3; and SI, Table S3).

When using artificially intelligent nanoarray based DFA analysis with leave-one-out cross-validation, each molecular sub-type of BC could be discriminated from other sub-types of cancer with accuracy of 81–88% for Luminal A, 78–86% for Luminal B and 83–90% for Triple negative sub-molecular classification. Sensitivities were in the range of 80% for the aforementioned molecular sub-types (Table 3; and SI, Table S3). ROC analysis of CV1 gave an ROC-AUC of 0.83–0.87 for Luminal A, 0.83–0.84 for Luminal B and 0.87–0.91 for triple negative sub-molecular classification. Furthermore, the molecular sub-types, Luminal A and Luminal B were successfully discriminated with sensitivities of 86–94%, specificities of 75–92% and accuracy range between 82 and 95% (Table 3; and SI, Table S3). Using artificially intelligent nanoarray based on DFA analysis, one could discriminate on the basis of hormone receptor status (luminal *vs*. non-luminal) with 70–88% sensitivity range, 71–87% specificity range, and accuracy between 71% and 88% (Table 3; and SI, Table S3). As before, HER2 status seems to be reflected in VOC levels in discriminating across the whole cohort, or within either luminal (86–100% accuracy) or non-luminal subtypes (91% accuracy), i.e. independently of hormonal status. Notably, when cases with equivocal HER status (HER2++ on IHC, but with no FISH data available) were tested using HER status DFA (based on unequivocal status), 35% were identified as HER2+, in keeping with the data previously reported for FISH testing of equivocal cases (Table 3; and SI, Table S3) [34, 35].

It is important to note that, when applying the same DFA model calculated in the different binary comparisons to distinguish between different confounding factors (e.g. the day the sample was taken, age, day of run, etc.), the volatolomic signatures of the 2 groups totally overlapped (refer to SI Section S2 and SI, Figure S1 for more information).

## DISCUSSION

GC-MS results showed detectable differences in breath VOCs between patients carrying tumours with different molecular signatures. VOCs detected in the breath of BC patients were from the families of alkanes and methylated alkanes, alcohols, aldehydes, ketones, chlorinated alkanes, acetic acid derivatives, benzene derivatives and ethers. The same families of VOCs were reported previously both in the breath and headspace of breast-derived cell lines, e.g. alkanes, ketone and halogenated hydrocarbons [36, 37]. In particular, Lavra *et al*. [37] showed that 2-ethyl-1-hexanol associated with breast cancer cell lines was significantly different, based on replication time and molecular markers of these lines. We find the same compound to be significantly different in the breath of healthy volunteers compared to patients carrying different breast disorders (*viz.* cancer and benign tumours) [37]. This may imply the potential of 2-ethyl-1-hexanol to serve as a biomarker for breast cancer proliferation and carcinogenesis. Moreover, some of the VOCs we detected are considered by products of DNA damage caused by enhance activity of ROS molecules in cancer cells. ROS molecules cause a chain reaction that eventually leads to oxidized purines and pyrimidines as well as strand breaks. ROS molecules may also enhance the activity of a large and diverse group of mixed oxidase enzymes (*i.e.* cytochrome p-450). Upregulation of CYP-450 enzymes in human breast tissue [38, 39], and especially aromatase, that synthesize oestrogens, are overexpressed in several types of breast cancers [40].

The majority of differences in metabolite profiles (and especially in methylated hydrocarbons) detected were between patients having breast disorders (malignant cancer and benign) and healthy controls. This may be due to the changes in metabolism and oxidative stress status between healthy people and people developing breast disorders. The same reason may also explain why the concentration of 7 compounds, among them heptane and heptane derivatives (products of oxidative stress), was below the limit of detection (LOD) in healthy states (SI, Table S2). It is also important to note that dimethyl ester carbonic acid was under the LOD for healthy volunteers, benign states, HER2, Luminal A and Luminal B sub-classification, but was found to have discriminatory power for HER2+ comparisons when the DFA model was applied (Table 2; and SI, Table S2). Another important enzyme is cytochrome P450 2E1 (CYP2E1), known to be overexpressed in breast cancer cells and to enhance ROS production [41]. It is induced by ethanol, which was elevated in breast diseases compared to healthy or benign conditions (SI, Table S1). Nevertheless, the relationship between ROS-induced VOC production and other metabolic pathways that contribute to VOC release from cancer cells is complex and has not been extensively studied in relation to molecular sub-types of breast cancer [41–43]. Moreover, no detectable change in specific volatiles was found for Luminal A or B sub-types, which may be due to its low proliferation status causing smaller differences in specific VOCs profiles. The changes could also be very small and undetectable by the method we used, which relies on Tenax tubes. This VOC capture process may alter VOC profile due to differential specificity of certain compounds to the Tenax sorbent [12, 18].

Our findings demonstrate that when multiple signals from artificially intelligent nanoarray are integrated into a single DFA value (*viz.* CV1), much more accurate identification is achieved compared to single compound analysis or even DFA analysis of the GC-MS output (*viz.* specific compound identification). Artificially intelligent nanoarrays discriminate between healthy or benign states and patients having BC with 88% accuracy in the training set and 83% in a blind test. Similar results come from Shuster *et al*. [25], which showed the ability of sensor array to distinguish with high accuracy between patients with negative mammography, benign diseases and breast cancer [25]. The ability to detect BC patients using breath VOCs has now been reported on several occasions [28, 31, 36, 44]. Our study makes it possible to specify the molecular sub-types of the malignant lesions from breast cancer patients, rather than just distinguishing breast cancer from benign states. The collective response signals obtained from multiple sensors provide us with volatolomic signatures rather than specific VOC identification. Considering the complexity of BC tumours, it is quite intuitive using responses from a multi-sensor system in conjugation with pattern recognition methods as opposed to detecting specific VOCs, as done by GC-MS. Notably, artificially intelligent nanoarrays provide additional discrimination properties, e.g. between luminal and non-luminal status, and between liminal A and luminal B subtypes, both of which are clinical useful in treatment stratification. Further treatment stratification for BC is currently being tested based on HER2 status; in this case both GC-MS and artificially intelligent nanoarrays could provide some discrimination.

The increasing complexity of molecular phenotypes for breast and other cancer types now leads to similar complexity in treatment options. It remains shown that breath analysis as described here is capable of discriminating multiple breast cancer sub-types using a single analysis. However, the ability to discriminate current molecular subtypes from a single breath analysis (albeit with individual discriminant functions for each comparison) supports the idea that it is possible if the technology is robust enough to undertake the larger validation investigations required, and if more sophisticated discriminant data models can be established.

Guidelines exist for current methodologies for molecular sub-type identification [45, 46], and their clinical application in treatment stratification is well established; however they are dependent on suitable biopsy material. In contrast, breath testing is not invasive and does not rely on biopsy, so it can be used in situations where biopsies are not easily obtained. Hence breath testing is better suited to rapid point-of-care testing that may aid screening in a wider healthcare setting, or the follow-up of patients for recurrent disease. Currently, treatment stratification in the metastatic setting relies mainly on primary tumour status, as biopsies of secondaries are often difficult to obtain. Breath analysis is not dependent on the location of the metastatic disease; it therefore provides the possibility of screening for molecular sub-type in the cases where cancers have metastasised. The ability to confirm molecular sub-type by breath analysis in this advanced setting is an intriguing possibility, providing opportunities for more customised/personalised treatment. Nevertheless, breath analysis for disease detection continues to suffer from certain limitations, including the effect of confounding factors on breath VOCs, often the relatively low levels of cancer-related VOCs in the breath, and the limited ability of current analytical methods to the detect a number of VOCs. The lack of standardization between different studies also makes disease detection through breath analysis difficult to implement in the clinical practice.

In conclusion, the concept of using a volatolomic signature to detect BC molecular phenotypes may revolutionize screening and targeted therapy. However, this study should only be considered as a proof of concept, a larger population size needs to be studied for significant statistical results, and to address the possibility that more information might be gained from volatolomic signatures. These results would have a beneficial impact on clinical practice, implying the possibility of combining a routine breath test as a possible alternative to the annual mammography and may in future provide a promising tool not only for the detection of breast cancer, but also for its phenotypic profiling all in a single breath test.

## MATERIALS AND METHODS

### Patients and group description

Breath samples were collected from 276 female volunteers aged 21–74 (average age 47; median age 46; range of ages 21 to 74). Among them were 30 healthy women, 52 women with benign conditions, 25 with ductal carcinoma *in situ* (DCIS) and 169 with malignant lesions of the breast (Figure 2, Table 1). Inclusion criteria were: (1) Females aged 18 to 75 years with newly diagnosed untreated lesions; (2) their lesions had been confirmed by histological or pathological methods as being breast cancer or benign breast lesions. Specific molecular typing depends on the postoperative pathological and IHC results; (3) biochemical examination: blood urea nitrogen ≤ 1.25 × upper limit of normal, creatinine ≤ 1.25 × upper limits of normal, total bilirubin ≤ 1.5 × upper limit of normal, alanine aminotransferase and aspartate aminotransferase ≤ 2.5 × upper limit of normal; (4) no statistical difference in demography; (5) the subjects gave informed consent. Exclusion criteria were patients: (1) suffering from different chronic diseases, such as diabetes, coronary heart disease, renal insufficiency, rheumatoid; (2) suffering from local or systemic infection during the 2 weeks before breath sampling; (3) diagnosed with obvious obstruction in the lung ventilation; (4) who had received chemotherapy, surgery or interventional therapy; (5) with a history of other tumours. Full patient data is available in Table S4 of SI. All volunteers were recruited at the First Affiliated Hospital of Anhui Medical University, Hefei, China, the Anhui Province Hospital and the Maternal and Child Health Hospital of Anhui Medical University. For this study, the volunteers were instructed to fast state overnight until sample collection in the morning. None of them consumed tobacco and/or alcohol. Using a series of advanced instrumentations, 5 distinct types of BC were identified (Table 1). The sub-types were confirmed by IHC and FISH. Tumor estrogen/progesterone receptor (ER/PR) status was determined by IHC, according to the College of American Pathologists Protocol for the histopathology of all invasive and non-invasive carcinomas of the breast (Table 1) [45, 46]. HER2 status was determined according to the ASCO/CAP HER2 guideline (Table 1) [45]. Approval was obtained from the ethical committee of the Affiliated Hospital of Anhui Medical University, Hefei, China. All volunteers gave their written informed consent before breath collection.

### Breath collection

Collection of the exhaled breath samples from all the volunteers took place in the same environmental conditions using the same sampling procedure. Impurities were cleared from the inhaled air by a lung washout, which involved a 3 min inhalation through a mouthpiece with a filter cartridge on the inspiratory port (Eco Medics, Duerten, Switzerland), thus reducing the concentration of exogenous VOCs (Figure 1A) [22, 47]. Room samples were collected in the morning of each sampling day to minimize potential environmental (hospital) contaminants that might develop throughout the day. After the washout process, the volunteers inhaled to full lung capacity and exhaled slowly through the mouthpiece into a separate exhalation port against 10–15 cm H_2_O pressure [48]. This assured the closure of the vellum to eliminate contamination through nasal entrainment. Exhaled breath consists of respiratory dead space air that was exhaled first and filled into designated dead space bag (later discarded), followed by the alveolar air from the lungs (used as sample). The alveolar breath was put into a 0.75 litre Tedlar^®^ bag (Keika Ventures, LLC) (Figure 1A). Phenol and dimethylacetamide, known contaminants in Tedlar bags, were not found in the breath samples [49]. Two bags were collected per patient for the analysis by GC-MS and artificially intelligent nanoarrays (see below). However, due to technical issues, not all breath samples collected were run on the GC-MS (details given in Table 1). After breath collection, VOCs in the samples were trapped and pre-concentrated in 2-bed ORBOTM 420 Tenax^®^ TA sorption tubes for gas and vapour sampling (Figure 1B), which were specially treated (35/60 mesh; 100/50 mg; disposable, preconditioned and sealed supplied by Sigma-Aldrich, China) by pumping with a Schego membrane pump modified with a rotameter (Cole-Parmer) and defined for flows between 50–1000 ml/min. Each breath sample has been collected on a sorbent tube for 7 min at a flow rate of 100 ml/min. Tenax TA is not sensitive to humidity and shows low H_2_O trapping [50], which is emphasized here because exhaled breath consists mainly of nitrogen, oxygen, carbon dioxide, water vapour and inert gases [15, 16, 51]; however the effect of high and varying humidity in room air and breath sample proved insignificant. VOCs produced endogenously by metabolism are present in much smaller amounts in exhaled breath, and many diseases are manifested through very gentle and small changes in the levels of these breath VOCs [18, 22, 52, 53]. Moreover, breakthrough volume should be considered when 0.7 litre of breath sample is pumped through Tenax traps. However, most VOCs should not be influenced by breakthrough since according to the information from Sigma-Aldrich, the volumes for Tenax TA under the given conditions were usually >10 litres per gram of resin. The Tenax tubes were kept refrigerated at 4°C until they reached the Laboratory for Nanomaterial-Based Devices (Technion - IIT, Israel) for analysis. The maximum duration between collection and the analysis of the samples was 4 months was. It has been confirmed that several VOCs in the breath of lung cancer patients (decane, benzene, aldehydes and branched aldehydes) can be trapped and stored in the ORBO 420 Tenax^®^ TA sorption tubes for > 6 months if the samples are cooled. Amann et al. [53] offered a standardization of a breath collection route that might be accepted in the future [53].

### Analysis strategy of breath samples

This study was conducted in 3 parts (Figure 2). Discrimination between the various disease state and/or sub-types was carried out through: (i) specific chemical analysis of breath VOCs using GC-MS analysis; and (ii) volatolomic signature of breath VOCs obtained by applying DFA analysis for the collective response from artificially intelligent nanoarrays. In the first part of each approach, healthy volunteers and those with benign breast lesions were discriminated from subjects with malignant breast cancer. Those with DCIS were tested using the discriminant CV identified. In the second part, women with malignant BC were discriminated from one another according to the molecular sub-type of their tumour. In the third part, we tested the discrimination of HER2 status based on breath VOC signatures. Classification success rates of the binary problems were calculated through leave-one-out cross-validation of the computed DFA models. For this purpose, one sample in each binary test was excluded and the DFA model was calculated based on the remaining samples, viz. the training set. The test sample was projected onto the CV1 axis calculated using the training set. The test sample was then “blinded” against the DFA model to disguise its class affiliation. This was repeated for all possibilities of leaving out one sample, the left out sample being classified as true positive (TP), true negative (TN), false positive (FP) or false negative (FN) for the calculation of sensitivity, specificity and accuracy. For artificially intelligent nanoarray analysis of healthy and benign volunteers compared to breast cancer patients, blind tests were conducted in which one-third of the samples were excluded from the calculated DFA model, and then projected onto the model (of the training set) to calculate TP, TN, FP and FN.

### GC-MS analysis

Breath VOCs were analysed with a thermal desorption system (TD20; Shimadzu Corporation) combined with a QP2010 GC-MS instrument (Shimadzu Corporations), having an SLB-5ms capillary column (with 5% phenyl methyl siloxane; 30 m length; 0.25 mm internal diameter; 0.5 μm thicknesses; from Sigma-Aldrich). Helium (99.999%), with additional Agilent triple filter for oxygen and humidity for further purification, served as the carrier gas. The oven profile was programmed to: (a) 5 min at 35°C; (b) increasing the temperature at 5°C/min until 180°C had been reached; (c) increasing the temperature at 13.5°C/min until 290°C had been reached; and (d) holding at 290°C for 1 min. Molecular structure of breath VOCs was tentatively determined through spectral library matches (compounds library of the National Institute of Standards and Technology, USA) using the GC-MS post-run analysis program (version 2.53; Shimadzu Corporation, Duisburg, Germany) (Figure 1C). None of the VOCs had a Gaussian distribution, thus the non-parametric Wilcoxon/Kruskal-Wallis test was used to distinguish significant compounds (*p* < 0.05) expressing differential values between molecularly different breast cancer lesions using SAS JMP (version 8.0, SAS Institute Inc., Cary, NC). Sensitivities and specificities were also determined.

### Breath analysis using artificially intelligent nanoarray

Alveolar air was further analysed by artificially intelligent nanoarray based on either GNPs and/or SWCNT coated with different organic layer (Figure 1D). The metallic core of the GNP and the SWCNT served as the electrical core, whereas different organic coatings of the gold core or the nanotube provided unique recognition layers. The chemiresistors are cross-reactive, showing sensitivity to various analytes in different strengths, and thus can be tuned for a particular sensing application [23, 47, 54, 55]. Breath VOCs were thermally desorbed from the Tenax TA tubes by a bespoke stainless steel thermal desorption device in 270°C for 10 min under ambient conditions. The desorption device was thoroughly cleaned with ethanol between experiments. VOCs were evacuated into a stainless-steel test chamber of 100 cm^3^ containing forty different sensors mounted on a custom polytetrafluoroethylene circuit board placed inside. All sensors were fabricated and characterized in Prof. Haick's laboratories. Full information on the synthesis, fabrication and sensing principles of the artificially intelligent nanoarray is described elsewhere [22, 32, 33, 47, 56]. Each sensor in the array responded in a different, reversible, time-dependent change in electrical resistance when exposed to breath VOCs. In a typical experiment, electrical signals were collected for 5 min vacuum (i.e. baseline), followed by 5 min of breath inside the chamber, and followed by another 5 min of vacuum to return to baseline. The responses from all sensors as a function of time were measured simultaneously by an Agilent Multifunction switch 34980. Collective responses from the reservoir of 40 sensors were analysed using a statistical pattern recognition algorithm (e.g. DFA). DFA is a supervised linear pattern recognition method used for data classification aimed at detecting the most significant read-outs from the collective responses of the sensors, and to create new space of canonical variable (CVs), such that the variance between the pre-defined classes is maximized, whereas variance within each class is minimized [57]. Canonical variables are obtained in manually orthogonal dimensions, thus effectively reducing the dimensions of the experimental data. Moreover, using the optimal cut-off of ROC based on CV1, the area under curve (ROC-AUC) was calculated. *P* values for the CV's, which were not normally distributed, were also determined by non-parametric Wilcoxon/Kruskal-Wallis tests using SAS JMP (version 8.0, SAS Institute Inc., Cary, NC). Values are usually presented in the form of a range since two independent experiments were involved in obtaining the volatolomic signatures of BC molecular sub-types by artificially intelligent nanoarray.

## SUPPLEMENTARY MATERIALS FIGURE AND TABLES


