# Supplementary material for: Differentiation between genetic mutations of breast cancer by breath volatolomics
Source: Oncotarget. 2015 Nov 2;6(42):44864–76. doi: 10.18632/oncotarget.6269 (PMC4792597; doi:10.18632/oncotarget.6269)
Supplement: Supplementary file 1 [file oncotarget-06-44864-s001.pdf]

Differentiation between genetic mutations of breast cancer by breath volatolomics

Supplementary Materials

Calibration of volatile organic compounds

Confirmation and quantification of compounds identified by GC-MS were achieved through calibration curves of external standards of pure materials purchased from Sigma-Aldrich (Table S1,). Calibration gas was inserted into a glass vial that had been thoroughly cleaned between different gases. Standards gases were produced using a commercial permeation/diffusion tube dilution (PDTD) system (Umwelttechnik MCZ, Germany). A constant flow of 200 ± 1 cm^3/min of dry pure nitrogen (99.999%) from a commercial nitrogen generator (N-30, On Site Gas Systems, USA) was mixed with a constant mass flow of vaporized VOC(s) exiting a diffusion tube (Dynacal, VICI Metronics). The nitrogen/VOC mixture exiting the PDTD system was further diluted with N_2 to concentrations in the range of a few ppb to hundreds of ppb. Control of the mass flow rate of the vaporized VOC(s) (via the temperature of the diffusion tubes) and the total volumetric nitrogen flow rate determines the VOC concentration. Two-bed ORBO™ 420 Tenax® TA sorption tubes were placed on the exit of the PDTD system to absorb the calibration gas in the same manner as the collection bags had been pumped.

VOCs contributing to discrimination in the different binary comparisons are from the families of branched and straight hydrocarbons, unsaturated hydrocarbons, alcohols, ketones, cyclic hydrocarbons (straight and branched), carbonate esters and benzene derivatives (Table S1). Levels of VOCs are given in Table S2 and are in the range of tens to hundreds ppb for ketones and alcohols (except for 6-methyl-5-heptene-2-one, which is in few ppbs), few to tens ppbs for esters and benzene derivatives, and sub-ppb for the other compounds.

Effect of confounding factors

To eliminate potential bias in results, we divided the groups according to confounding factors based on age and date of sampling. For this, the breath samples were grouped according to patient age (below or above 50 years) and date on which they were taken. DFA models were plotted onto the divided groups. This was done for every binary comparison and DFA model calculated. To characterise the effect of the potential confounding factor, the ROC-AUC of the calculated CV1 was used. All the women were not smokers, thus gender and smoking status was not under test. Representative results of DFA models based on age are shown in Figure S1, which shows that the ROC-AUC of the tested DFA models was < 0.67, from which it can be concluded that none of the factors significantly influenced the results.

Table S1. List of 23 VOCs identified by the GC-MS to show significant difference between room and breath samples ($p < 0.05$). *X* = Mann Whitney with significance at $p < 0.05$ for test stated (groups given in Table1); *ns* = non-significant, *vs* = versus. No compound showed a significant trend for detecting Luminal A or B *vs.* other cancers, or discriminating between Luminal A and Luminal B

Suspected VOC	Healthy *vs.* Benign + Cancer	Healthy + Benign *vs.* Cancer	Cancer *vs.* DCIS	Triple Negative *vs.* Other	HER2+ *vs.* Other	HER2 + status* *vs.* Other	HER2+ status* (Non Luminal) *vs.* Other	HER2+ status* (Luminal) *vs.* Other
Ethanol	X	X	X	ns	ns	X	ns	ns
Acetone	X	ns	ns	ns	ns	ns	ns	X
Cyclopentane	X	X	ns	ns	ns	ns	ns	ns
Pentane, 3-methyl-	X	X	ns	ns	ns	ns	ns	X
Ethyl Acetate	X	X	X	ns	X	ns	X	ns
Carbonic acid, dimethyl ester (DMC)	X	X	ns	X	X	X	X	ns
Cyclopentane, methyl-	X	X	ns	ns	X	X	X	ns
Pentane, 2, 3-dimethyl-	X	X	ns	ns	ns	ns	ns	ns
Heptane	ns	X	ns	ns	X	X	ns	X
Toluene	X	X	X	ns	ns	ns	ns	ns
Cyclohexane, 1, 4-dimethyl-	ns	ns	ns	X	ns	ns	X	ns
Hexanal	X	X	ns	ns	ns	ns	ns	ns
Acetic acid, butyl ester	X	X	ns	X	ns	ns	X	ns
Heptane, 2, 4-dimethyl-	X	X	ns	ns	ns	ns	ns	ns
Ethylbenzene	X	X	ns	ns	ns	ns	ns	ns
Benzene, 1, 3-dimethyl-	X	X	ns	ns	X	ns	X	ns
2-Propenoic acid, butyl ester	X	X	ns	ns	ns	X	X	ns
Styrene	X	ns	X	ns	ns	ns	X	ns
Heptane, 2, 3, 4-trimethyl-	X	ns	ns	ns	ns	ns	ns	ns
Benzene, (1-methylethyl)-	X	ns	ns	ns	ns	ns	X	ns
alpha.-Pinene	X	ns	ns	ns	ns	ns	ns	ns
5-Hepten-2-one, 6-methyl-	X	ns	ns	ns	ns	ns	ns	ns
1-Hexanol, 2-ethyl-	X	ns	ns	ns	ns	ns	ns	ns

*HER2+ status related VOC identified based on HER2 negative (IHC− or −/+) vs. HER2+ (IHC+++ or IHC++/FISH+).

Table S2. Averaged values (as ppb) and their standard deviation (SD) for the 23 VOCs identified by GC-MS to have significant difference between breath samples ($p < 0.05$). Mass to ion ration (m/z), retention time (RT), linear regression (R^2), limit of detection (LOD) and limit of quantification (LOQ) are given

VOC	m/z	RT [min]	R2	LOD [ppb]	LOQ [ppb]	Healthy Average [ppb]	Healthy STDEV [pbb]	Benign Average [ppb]	Benign STDEV [pbb]	DCIS Average [ppb]	DCIS STDEV [pbb]
**Ethanol**	31	2.36	0.99	7.78	25.9	209	215	458	132	917	118
**Acetone**	43	2.55	0.99	0.766	2.55	154	253	268	93.7	350	411
**Cyclopentane**	42	3.7	0.95	0.025	0.082	0.096	0.248	0.584	0.228	1.39	1.93
**Pentane, 3-methyl-**	57	3.96	0	0.02	0.079	0.11	0.151	0.102	0.109	0.181	0.334
**Ethyl Acetate**	43	4.65	1	0.04	0.141	0.161	0.326	0.41	0.364	1.52	2.55
**carbonic acid, dimethyl ester**	45	4.68	0.99	0.289	0.966	< LOD	< LOD	< LOD	< LOD	0.469	0.969
**Cyclopentane, methyl-**	56	5.14	0.99	0.256	0.856	< LOD	< LOD	0.487	0.44	1.95	3.93
**Pentane, 2,3-dimethyl-**	43	6.55	0.96	3.56	11.86	0.411	1.44	0.441	1.05	0.243	0.751
**Heptane**	43	8.1	1	0.008	0.03	< LOD	< LOD	0.111	0.187	0.231	0.571
**Toluene**	91	12.8	0.99	0.07	0.234	1.87	4.93	12.8	10.3	36.6	51.1
**Cyclohexane, 1,4-dimethyl-**	55	25	0.98	0.031	0.103	0	0.001	0	0.001	0.023	0.072
**Hexanal**	44	15.5	0.99	0.01	0.048	0.014	0.045	< LOD	< LOD	0.104	0.11
**Acetic acid, butyl ester**	43	13.4	0.94	0.031	0.103	0.109	0.25	0.783	0.427	1.73	3.1

VOC	HER2equiv (non-luminal) Average [ppb]	HER2equiv (non-luminal) STDEV [pbb]	Luminal A Average [ppb]	Luminal A STDEV [pbb]	Luminal B Average [ppb]	Luminal B STDEV [pbb]	Triple Negative Average [ppb]	Triple Negative STDEV [pbb]
**Ethanol**	480	339	336	205	400	198	397	145
**Acetone**	198	121	281	196	255	243	176	75.4
**Cyclopentane**	1.09	2.86	1.88	4.56	4.59	9.02	5.05	7.23
**Pentane, 3-methyl-**	0.185	0.282	0.227	0.348	0.435	0.735	0.481	0.683
**Ethyl Acetate**	0.561	1.05	0.796	1.23	1.05	1.74	2.08	2.52
**carbonic acid, dimethyl ester**	< LOD	< LOD	< LOD	< LOD	< LOD	< LOD	0.359	0.318
**Cyclopentane, methyl-**	0.717	1.38	1.08	1.65	1.65	3.48	2.41	2.92
**Pentane, 2,3-dimethyl-**	0.876	2.27	0.712	1.40	0.978	1.48	1.75	1.69
**Heptane**	0.122	0.357	0.197	0.412	0.266	0.51	0.622	0.931
**Toluene**	15.3	18.7	18.9	20.9	23.0	24.4	36.0	40.7
**Cyclohexane, 1,4-dimethyl-**	0.006	0.029	0	0.002	0.012	0.036	0.032	0.062
**Hexanal**	0.144	0.268	0.159	0.323	0.322	0.503	0.591	0.796
**Acetic acid, butyl ester**	0.781	0.984	0.591	0.982	0.773	0.994	2.27	2.66

**Heptane, 2, 4-dimethyl-**	43	18	0.99	0.01	0.035	< LOD	< LOD	0.023	0.02	0.078	0.11	0.04	0.09	0.05	0.1	0.09	0.18	0.13	0.22
**Ethylbenzene**	91	19	0.92	0.023	0.078	0.259	1.09	1.99	3.36	9.52	20.3	3.86	6.99	6.80	8.61	6.83	12.2	13.7	17
**Benzene, 1, 3-dimethyl-**	91	19.4	0.92	0.0556	0.185	0.548	1.45	4.38	3.45	9.03	8.43	3.91	5.21	6.24	4.87	6.69	5.70	9.18	8.75
**2-Propenoic acid, butyl ester (*)**	55	31.4	0.94	0.0192	0.064	1.5	4.1	10.4	8.1	27.7	34.3	10.9	10.4	9.7	9.4	13.8	13.5	22.1	18.9
**Styrene**	104	15	0.91	0.0048	0.0158	7.94	20.9	53.9	41.7	143	177	56.6	54.0	50.2	48.5	71.3	69.9	114	97.7
**Heptane, 2, 3, 4-trimethyl-**	57	6.53	0.99	0.005	0.015	< LOD	< LOD	0.005	0.007	< LOD	< LOD	0.011	0.03	< LOD	< LOD	0.02	0.05	0.05	0.07
**Benzene, (1-methylethyl)-**	105	27.5	0.98	0.362	1.21	0.394	1.07	3.42	2.68	10.6	16.1	4.09	5.58	5.00	7.35	7.15	9.97	12.5	14.6
**alpha.-Pinene**	93	22.7	0.9	0.0018	0.0059	0.023	0.058	0.127	0.112	0.376	0.538	0.232	0.441	0.17	0.217	0.263	0.297	0.403	0.443
**5-Hepten-2-one, 6-methyl-**	43	25.2	0.99	1.88	3.47	< LOD	< LOD	3.78	3.21	7.63	8.93	3.59	2.80	3.85	4.38	4.30	3.23	5.42	3.82
**1-hexanol, 2-ethyl**	57	20	0.93	0.03	0.1	3.07	5.56	15.3	15.2	25.3	10.7	15.5	18.3	16.3	18.6	20.4	20.0	29.4	29.6

*calibrated by butyl acetate

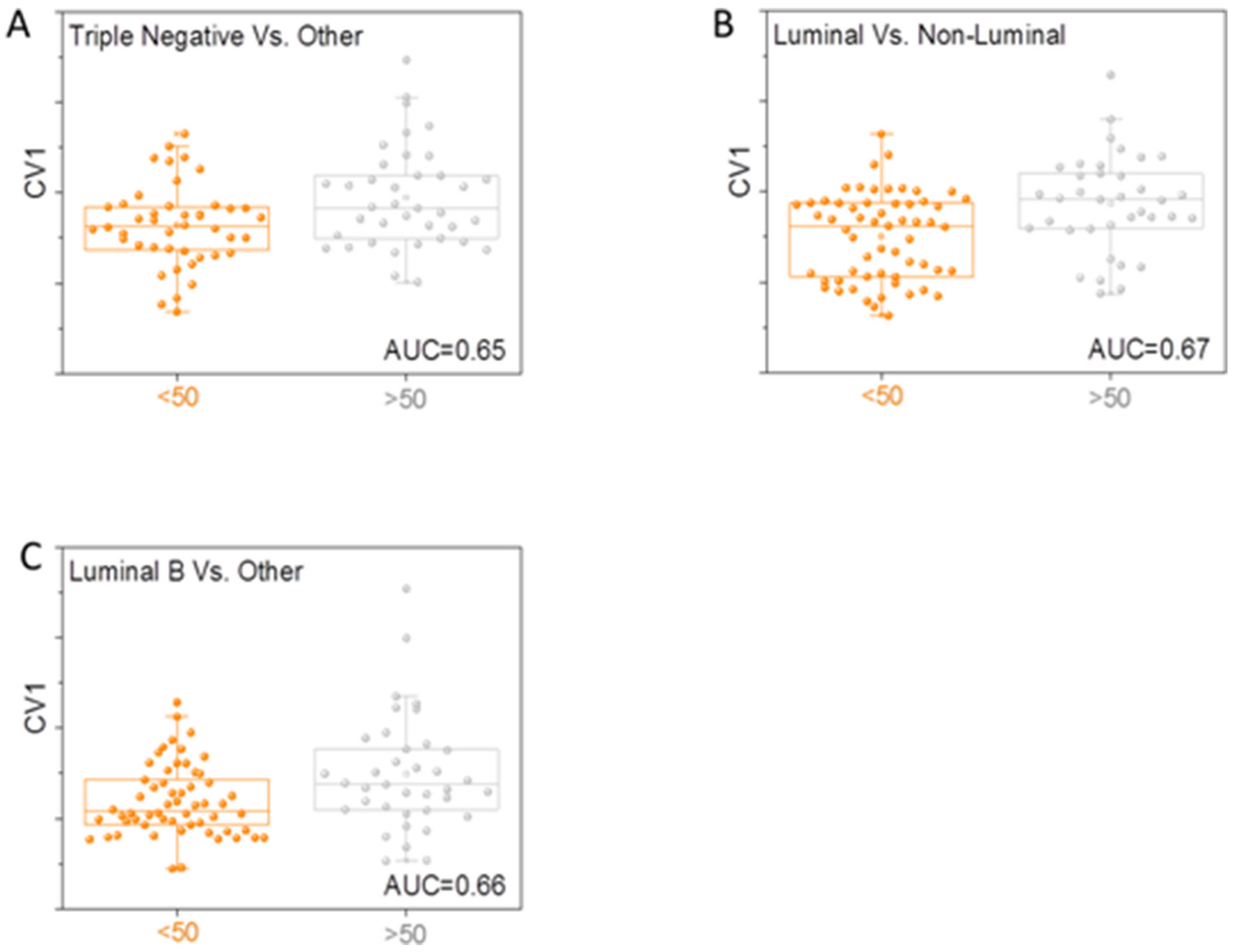

Supplementary Figure S1. Representative DFA plots of CV values obtained from the response of the sensor array to breath VOCs of different sub-groups divided into 2 groups; above and below 50 years old. Comparisons are shown for (**A**) breast cancer patients with triple negative molecular sub-type compared to all other sub-types; (**B**) breast cancer patients with luminal molecular sub-type compared to non-luminal BC; and (**C**) for Luminal B molecular sub-type compared to all other sub types of breast cancer patients. Values of ROC-AUC for the calculated CV are presented for each DFA model.

Table S3. Table representing the 2 batches in this study and the sample size of each comparison. Classification success calculated for the CV values obtained from DFA analysis of the sensor array responses represented for 2 independent studies (Groups A and B). Sensitivity, specificity, accuracy and AUC were calculated according to the confusion matrix of each study separately, and averages are given. *P* values for the CV's were also determined by Wilcoxon/Kruskal-Wallis tests. ND = not done

Comparison	Group A						Group B					
	Sample size	Accuracy [%]	Sensitivity [%}	Specificity [%]	*P* value	AUC	Sample size	Accuracy [%]	Sensitivity [%]	Specificity [%]	*P* value	AUC
Healthy+Benign *vs.* Cancer	0	ND	ND	ND	ND	ND	140	87.9	82.2	90.6	< 0.0001	0.91
Benign *vs.* Cancer	110	71.2	62.2	75.7	< 0.0001	0.73	111	82	80	82.3	< 0.0001	0.82
Cancer *vs.* DCIS	85	81.4	83.3	81.1	0.0006	0.81	109	84.4	83	92	< 0.0001	0.89
Luminal A *vs.* Other	73	87.7	87.5	87.5	0.0025	0.83	96	81.3	75	82.1	< 0.0001	0.87
Luminal B *vs.* Other	73	86.3	85.3	87.2	< 0.0001	0.84	96	78.1	83.3	74.1	< 0.0001	0.83
Triple Negative vs. Other	62	90.3	93.3	89.4	< 0.0001	0.91	82	82.9	83.3	82.9	< 0.0001	0.87
HER2+ vs. Other	68	82.4	91	80.7	0.0003	0.85	96	81.3	81.3	81.3	< 0.0001	0.86
HER2+ status*	48	95.8	100	95.1	< 0.0001	0.99	62	80.7	77.8	82.9	< 0.0001	0.85
HER2+ status* (Non-Luminal)	0	ND	ND	ND	ND	ND	22	90.9	90.9	90.9	0.0007	0.93
HER2+ status* (Luminal)	20	100	100	100	0.0011	1	28	85.7	87.5	83.3	0.16	0.66
Luminal *vs.* Non-Luminal	73	87.7	88.1	87.1	< 0.0001	0.86	96	70.8	70.4	71.4	0.0043	0.67
Luminal A *vs.* Luminal B	42	85.7	75	88.2	0.0014	0.87	56	94	91.7	95.2	< 0.0001	0.96

Table S4: Full patients clinical data including; age; menopause status;tumor grade; TNM stage; ER, PgR and HER2 IHC results; HER2 FISH results; Ki67 IHC results; and other IHC results

Category	Age	Post-Meno-pause	grade	TNM stage	ER IHC	PgR IHC	HER2 IHC	HER2 FISH	Ki67 IHC	Other IHC results
LuminalA	43	no	2		pos	pos	neg (−)		0	E-cad (+), EGFR (−)
LuminalA	57	yes	2	I	pos	pos	neg (−)		3%	CK5/6 (−), calponin (−)
LuminalA	44	no		IIA	pos	pos	neg (−)		5%	CK5/6 (−), EGFR (−)
LuminalA	48	no			pos	pos	neg (−)		5%	CK5/6 (−), EGFR (−)
LuminalA	63	yes	3	IIIA	pos	neg	neg (−)		7%	CK5/6 (−), EGFR (−), E-cad (+)
LuminalA	36	no			pos	pos	neg (−)		7%	CK5/6 (−), calponin (−), P63 (−), CD10 (−)
LuminalA	42	no	2	IIA	neg	pos	neg (−)		8%	CK5/6 (−), EGFR (−)
LuminalA	39	no		I	pos	pos	neg (−)		10%	CK5/6 (−), EGFR (−)
LuminalA	61	yes	2	IIIC	pos	neg	neg (−)		10%	CK5/6 (−), EGFR (−)
LuminalA	59	yes	2	IIB	pos	pos	neg (−)		10%	CK5/6 (−), EGFR (−), E-cad (+)
LuminalA	55	no	2	IIIA	pos	neg	neg (−)		10%	CK5/6 (−), EGFR (−)
LuminalA	47	no	2	IIA	pos	pos	neg (1+)		3%	E-cad (−), EGFR (−)
LuminalA	34	no		I	pos	pos	neg (1+)		3%	CK5/6 (−), calponin (−)
LuminalA	38	no	2	IIA	pos	pos	neg (1+)		5%	CK5/6 (−), EGFR (−), CD10 (−)
LuminalA	47	no	2		pos	pos	neg (1+)		5%	CK5/6 (−), EGFR (−)
LuminalA	50	no	2	I	pos	pos	neg (1+)		5%	CK5/6 (−), EGFR (−), E-cad (+)
LuminalA	48	no	2		pos	pos	neg (1+)		7%	CK5/6 (−), EGFR (−), P120 (+)
LuminalA	51	no	2		pos	pos	neg (1+)		8%	E-cad (+), EGFR (−)
LuminalA	60	yes		IIA	pos	pos	neg (1+)		10%	CK5/6 (−), E-cad (+), P120 (+), EGFR (−)
LuminalA	69	no	2	I	pos	neg	neg (1+)		10%	CK5/6 (−), EGFR (−)
LuminalB	41	no	2	IIA	pos	pos	equiv (2+)		5%	CK5/6 (−), EGFR (−)
LuminalB	31	no			pos	neg	equiv (2+)		10%	CK5/6 (−)
LuminalB	43	no	2	IIA	pos	pos	equiv (2+)		15%	
LuminalB	31	no	3	IIA	pos	pos	equiv (2+)		15%	EGFR (−), P120 (+), E-cad (+)
LuminalB	41	no	2		pos	pos	equiv (2+)		15%	
LuminalB	40	no	2		pos	pos	equiv (2+)		15%	CK5/6 (−), EGFR (−), P120 (+), E-cad (+), calponin (−), p63 (−), CD10 (−), SMA (−)
LuminalB	41	no	2		pos	pos	equiv (2+)		15%	CK5/6 (−), EGFR (−), E-cad (+), calponin (−)

LuminalB	49	no	2	IIA	pos	pos	equiv (2+)		15%	CK5/6 (−), EGFR (−)
LuminalB	38	no	2	I	pos	neg	equiv (2+)		15%	CK5/6 (−), EGFR (−)
LuminalB	50	yes	2	IIB	pos	pos	equiv (2+)		15%	CK5/6 (−), EGFR (−), calponin (−), p63 (−)
LuminalB	51	no			pos	pos	equiv (2+)		20%	CK5/6 (−), EGFR (−),
LuminalB	62	yes	2		pos	pos	equiv (2+)		20%	CK5/6 (−), EGFR (−),
LuminalB	45	no	2		pos	pos	equiv (2+)		20%	CK5/6 (−), EGFR (−),
LuminalB	60	yes	2	IIIA	pos	pos	equiv (2+)		25%	P120 (+), EGFR (−)
LuminalB	61	yes		IIB	pos	neg	equiv (2+)		30%	CK5/6 (+), EGFR (−)
LuminalB	46	no	3	IIIA	pos	pos	equiv (2+)		30%	SMA (−)
LuminalB	56	no			pos	pos	equiv (2+)		30%	ER (+80CK5/6 (−), E-cad (+), EGFR (−)
LuminalB	38	no	3	IIA	pos	pos	equiv (2+)		30%	CK5/6 (−), E-cad (+), EGFR (−)
LuminalB	46	no			pos	pos	equiv (2+)		30%	CK5/6 (−), E-cad (+), EGFR (−)
LuminalB	44	no			pos	pos	equiv (2+)		30%	CK5/6 (−), EGFR (−)
LuminalB	50	no			pos	pos	equiv (2+)		30%	
LuminalB	50	no	2	IIIA	pos	neg	equiv (2+)		35%	CK5/6 (−), EGFR (part +)
LuminalB	49	no	3	IIB	pos	pos	equiv (2+)		35%	CK5/6 (−), EGFR (−)
LuminalB	56	yes	2		pos	pos	equiv (2+)		40%	CK5/6 (−), EGFR (−), P120 (+)
LuminalB	52	yes	2	IIA	pos	neg	equiv (2+)		40%	CK5/6 (−), EGFR (−)
LuminalB	42	no			pos	pos	equiv (2+)		40%	EGFR (−), P120 (+), E-cad (+)
LuminalB	64	yes	2	IIB	pos	neg	equiv (2+)		40%	
LuminalB	40	no	2		pos	pos	equiv (2+)		60%	CK5/6 (−), EGFR (−)
LuminalB	56	no	2		pos	pos	equiv (2+)		80%	EGFR (−), P120 (+), E-cad (+)
LuminalB	45	no	2		pos	pos	equiv (2+)		90%	CK5/6 (−), EGFR (−)
LuminalB	41	no	2		pos	pos	equiv (2+)		90%	CK5/6 (−), EGFR (−)
LuminalB	44	no	2	IIA	pos	pos	neg (−)		15%	CK5/6 (−), EGFR (−)
LuminalB	47	no			pos	pos	neg (−)		15%	CK5/6 (−)

LuminalB	51	no	2	I	pos	pos	neg (−)		18%	CK5/6 (−), EGFR (−)
LuminalB	44	no	2	IIB	pos	pos	neg (−)		20%	CK5/6 (−), EGFR (−)
LuminalB	40	no	2	IIIC	pos	pos	neg (−)		20%	CK5/6 (−), E-cad (+), EGFR (−)
LuminalB	50	no		IIIC	pos	neg	neg (−)		20%	CK5/6 (−)
LuminalB	48	no	2	IIA	pos	pos	neg (−)		20%	CK5/6 (−), EGFR (−)
LuminalB	65	yes		IIA	pos	neg	neg (−)		20%	CK5/6 (−), EGFR (−), P120 (+), E-cad (−)
LuminalB	48	no	2		pos	pos	neg (−)		20%	E-cad (+), EGFR (−)
LuminalB	55	yes	2	IIA	pos	pos	neg (−)		20%	CK5/6 (−), EGFR (−)
LuminalB	49	no	3	IIB	pos	neg	neg (−)		30%	CK5/6 (−), EGFR (−)
LuminalB	66	yes	2	IIIA	pos	pos	neg (−)		30%	EGFR (−), P120 (+), E-cad (+)
LuminalB	46	no	2		pos	pos	neg (−)		35%	CK5/6 (−), E-cad (+), EGFR (−)
LuminalB	64	yes		IIA	pos	neg	neg (−)		35%	CK5/6 (−), EGFR (−)
LuminalB	47	no			neg	pos	neg (−)		40%	CK5/6 (−), EGFR (−), P120 (+), E-cad (+)
LuminalB	62	yes		IIA	pos	pos	neg (1+)		15%	EGFR (−), CK5/6 (−), Syn (−), CgA (−)
LuminalB	34	no			pos	pos	neg (1+)		15%	CK5/6 (−), EGFR (−)
LuminalB	42	no	2	IIA	pos	pos	neg (1+)		20%	CK5/6 (−), EGFR (−)
LuminalB	62	yes			pos	neg	neg (1+)		20%	CK5/6 (−), E-cad (+), EGFR (−)
LuminalB	43	no	2	IIA	pos	pos	neg (1+)		20%	CK5/6 (−), EGFR (−), P120 (+), E-cad (+), calponin (−), p63 (−), CD10 (−), SMA (−)
LuminalB	37	no	2		pos	pos	neg (1+)		25%	CK5/6 (−), EGFR (−)
LuminalB	69	yes		IIB	pos	pos	neg (1+)		30%	EGFR (−)
LuminalB	51	no	2	IIA	pos	pos	neg (1+)		30%	EGFR (−)
LuminalB	62	yes	3	IIA	pos	pos	neg (1+)		30%	CK5/6 (−), E-cad (+) EGFR (−)
LuminalB	45	no			pos	pos	neg (1+)		40%	CK5/6 (−), EGFR (−)
LuminalB	43	no	2	IIIC	pos	pos	neg (1+)		40%	CK5/6 (−), EGFR (−)
LuminalB	48	no	2		pos	pos	neg (1+)		40%	CK5/6 (−), EGFR (−)
LuminalB	35	no	2	IIB	pos	pos	neg (1+)		40%	CK5/6 (−), EGFR (−), P120 (+), E-cad (+)
LuminalB	57	yes	2	IIA	pos	pos	neg (1+)		45%	CK5/6 (−), EGFR (−)
LuminalB	45	no	3	IIA	pos	neg	neg (1+)		50%	CK5/6 (+), EGFR (+)
LuminalB	41	no	2	IIA	pos	pos	neg (1+)		50%	EGFR (−), P120 (+), E-cad (+), CK5/6 (−)
LuminalB	39	no	2	IIIC	pos	pos	neg (1+)		60%	CK5/6 (−), EGFR (−)
LuminalB	44	no	3	I	pos	neg	neg (1+)		75%	CK5/6 (+), EGFR (+)
LuminalB	68	no	2	IIA	pos	neg	pos (3+)		15%	CK5/6 (−), EGFR (−)
LuminalB	25	no	2	IIIC	pos	pos	pos (3+)		20%	CK5/6 (−), E-cad (+), EGFR (−)

LuminalB	51	no	2		pos	pos	pos (3+)		20%	
LuminalB	61	yes	2	IIB	pos	neg	pos (3+)		25%	CK5/6 (−), E-cad (+) EGFR (+)
LuminalB	53	no			pos	pos	pos (3+)		30%	
LuminalB	46	no		IIB	pos	pos	pos (3+)		30%	CK5/6 (−), EGFR (−)
LuminalB	59	no	3	IIB	pos	pos	pos (3+)		35%	CK5/6 (−), EGFR (−)
LuminalB	47	no			pos	pos	pos (3+)		35%	CK5/6 (−), EGFR (−)
LuminalB	48	no			pos	neg	pos (3+)		40%	CK5/6 (−), E-cad (+)
LuminalB	57	no			pos	pos	pos (3+)		40%	CK5/6 (−), E-cad (+)
LuminalB	47	no	2	IIIA	pos	pos	pos (3+)		40%	CK5/6 (−), E-cad (+), EGFR (−)
LuminalB	50	no	2	IIIA	pos	pos	pos (3+)		40%	CK5/6 (−), EGFR (−)
Triple Neg	42	no	2		neg	neg	neg (−)		5%	CK5/6 (−), EGFR (−)
Triple Neg	65	yes	2		neg	neg	neg (−)		10%	ER (−), PR (−), HER−2 (−), Ki−67 (+, 10%)
Triple Neg	34	no			neg	neg	neg (−)		10%	ER (−), PR (−), HER−2 (−), Ki−67 (+, 10%), E-cad (+), EGFR (−)
Triple Neg	42	no			neg	neg	neg (−)		15%	
Triple Neg	45	no	2		neg	neg	neg (−)		15%	
Triple Neg	60	yes			neg	neg	neg (−)		30%	CK5/6 (−),
Triple Neg	41	no	2		neg	neg	neg (−)		30%	CK5/6 (−), EGFR (−)
Triple Neg	48	no	3	IA	neg	neg	neg (−)		35%	E-cad (+), EGFR (−)
Triple Neg	44	no	2		neg	neg	neg (−)		40%	CK5/6 (+), EGFR (−)
Triple Neg	51	yes	3		neg	neg	neg (−)		40%	CK5/6 (+), EGFR (−)
Triple Neg	31	no	3		neg	neg	neg (−)		50%	E-cad (+),
Triple Neg	39	no		IIA	neg	neg	neg (−)		50%	EGFR (±), P120 (+)
Triple Neg	57	no	2		neg	neg	neg (−)		50%	CK5/6 (−), EGFR (−)
Triple Neg	57	yes			neg	neg	neg (−)		60%	CK5/6 (−), E-cad (−), CK7 (−),
Triple Neg	50	no		IIA	neg	neg	neg (−)		70%	E-cad (+), EGFR (±)
Triple Neg	21	no			neg	neg	neg (−)		75%	CK5/6 (+), EGFR (±)
Triple Neg	47	no			neg	neg	neg (−)		80%	CK5/6 (−), E-cad (+), EGFR (+)
Triple Neg	53	no	2	IIA	neg	neg	neg (−)		85%	CK5/6 (+), E-cad (+), EGFR (+), P120 (+)
Triple Neg	49	no	3	IIA	neg	neg	neg (1+)		20%	E-cad (+), EGFR (−)
Triple Neg	69	yes	2	IIA	neg	neg	neg (1+)		20%	E-cad (+), EGFR (−)
Triple Neg	41	no			neg	neg	neg (1+)		20%	E-cad (+), EGFR (−)
Triple Neg	51	no			neg	neg	neg (1+)		30%	CK5/6 (−), E-cad (+)
Triple Neg	57	yes	2		neg	neg	neg (1+)		40%	CK5/6 (−), E-cad (+), EGFR (+)
Triple Neg	52	no			neg	neg	neg (1+)		50%	E-cad (+), EGFR (−)
Triple Neg	63	yes		IV	neg	neg	neg (1+)		50%	E-cad (+), EGFR (−)
Triple Neg	68	yes	2	IIA	neg	neg	neg (1+)		60%	CK5/6 (−), E-cad (+)
Triple Neg	45	no			neg	neg	neg (1+)		50%	E-cad (+), EGFR (−)

HER2++	59	yes	3		neg	neg	equiv (2+)	pos	30%	CK5/6 (−), EGFR (−)
HER2++	50	yes	2	I	neg	neg	equiv (2+)	pos	30%	CK5/6 (−), EGFR (−)
HER2++	36	no		IV	neg	neg	pos (3+)	pos	5%	
HER2++	67	yes			neg	neg	pos (3+)	pos	15%	CK5/6 (−), EGFR (−)
HER2++	51	yes	3	IIA	neg	neg	pos (3+)	pos	15%	CK5/6 (−), EGFR (−)
HER2++	60	yes			neg	neg	pos (3+)	pos	20%	E-cad (+), P120 (+)
HER2++	48	no	2	IIIC	neg	neg	pos (3+)	pos	30%	CK5/6 (−), EGFR (−)
HER2++	53	no		IIA	neg	neg	pos (3+)	pos	30%	CD10 (−), P63 (−), Calponin (−)
HER2++	36	no	3	IIIA	neg	neg	pos (3+)	pos	30%	CK5/6 (−), EGFR (−)
HER2++	45	no	3	IIIA	neg	neg	pos (3+)	pos	30%	CK5/6 (−), EGFR (−)
HER2++	32	no	2	IIIC	neg	neg	pos (3+)	pos	30%	CK5/6 (−), EGFR (−)
HER2++	55	yes		IV	neg	neg	pos (3+)	pos	30%	CK5/6 (−), EGFR (−)
HER2++	63	yes		IV	neg	neg	pos (3+)	pos	30%	CK5/6 (−), EGFR (−)
HER2++	46	no		IV	neg	neg	pos (3+)	pos	30%	
HER2++	43	no	2		neg	neg	pos (3+)	pos	30%	CK5/6 (−), EGFR (−)
HER2++	46	no	2		neg	neg	pos (3+)	pos	30%	CK5/6 (−), EGFR (−), E-cad (+)
HER2++	59	yes		IIIC	neg	neg	pos (3+)	pos	40%	
HER2++	63	yes		IIIC	neg	neg	pos (3+)	pos	40%	
HER2++	47	no	3		neg	neg	pos (3+)	pos	50%	
HER2++	51	no	3	IIA	neg	neg	pos (3+)	pos	60%	CK5/6 (−), E-cad (+)
HER2++	38	no		IV	neg	neg	pos (3+)	pos	60%	
HER2 equivocal	59	yes	2	IIB	neg	neg	equiv (2+)	ND	15%	CK5/6 (−), EGFR (+), E-cad (+), P120 (+)
HER2 equivocal	53	no		IIIC	neg	neg	equiv (2+)	ND	20%	CK5/6 (−), EGFR (−), E-cad (+), P120 (+)
HER2 equivocal	56	no	2		neg	neg	equiv (2+)	ND	20%	ER (−), PR (−), HER−2 (2+), Ki−67 (+, 20%)
HER2 equivocal	57	yes	2		neg	neg	equiv (2+)	ND	20%	CK5 (−), EGFR (−), E-cad (+), P120 (+)
HER2 equivocal	51	no	3	IIB	neg	neg	equiv (2+)	ND	20%	CK5/6 (−), EGFR (−), E-cad (+)
HER2 equivocal	47	no			neg	neg	equiv (2+)	ND	20%	CK5/6 (−), EGFR (−), E-cad (+)
HER2 equivocal	51	yes		IIA	neg	neg	equiv (2+)	ND	20%	CK5/6 (−), S−100 (±), E-cad (−)
HER2 equivocal	45	no			neg	neg	equiv (2+)	ND	20%	
HER2 equivocal	50	no	2	IIIA	neg	neg	equiv (2+)	ND	30%	CK5/6 (−), EGFR (±), E-cad (+), P120 (+)
HER2 equivocal	46	no	3	IIA	neg	neg	equiv (2+)	ND	30%	
HER2 equivocal	57	yes	3	IIA	neg	neg	equiv (2+)	ND	30%	
HER2 equivocal	45	no	2	IIA	neg	neg	equiv (2+)	ND	35%	CK5/6 (−), E-cad (+), P120 (+)

HER2 equivocal	48	no	2	IIIA	neg	neg	equiv (2+)	ND	40%	CK5/6 (−), EGFR (+)
HER2 equivocal	55	yes	2	IIB	neg	neg	equiv (2+)	ND	40%	CK5/6 (−), EGFR (−)
HER2 equivocal	53	no	2	IIIC	neg	neg	equiv (2+)	ND	45%	CK5 (+), EGFR (+), E-cad (+), P120 (+)
HER2 equivocal	46	no	2	IIA	neg	neg	equiv (2+)	ND	50%	EGFR (+), E-cad (+), P120 (+)
HER2 equivocal	42	no			neg	neg	equiv (2+)	ND	55%	EGFR (−), E-cad (+)
HER2 equivocal	34	no	2	IIA	neg	neg	equiv (2+)	ND	60%	
HER2 equivocal	46	no			neg	neg	equiv (2+)	ND	60%	CK5/6 (−)
HER2 equivocal	53	no			neg	neg	equiv (2+)	ND	65%	E-cad (+), P120 (+)
HER2 equivocal	49	no		IIA	neg	neg	equiv (2+)	ND	70%	E-cad (+), P120 (+)
HER2 equivocal	45	no	3	IIA	neg	neg	equiv (2+)	ND	70%	P120 (+)
HER2 equivocal	63	yes		IIB	neg	neg	equiv (2+)	ND	70%	CK5/6 (−), EGFR (+), E-cad (+), P120 (+)
HER2 equivocal	56	yes	2		neg	neg	equiv (2+)	ND		
HER2 equivocal	61	yes	2	IIIC	neg	neg	equiv (2+)	ND		CK5/6 (−), EGFR (−), E-cad (+), P120 (+)
DCIS	41	no			neg	neg	pos (3+)		15%	CK5/6 (−), EGFR (−), E-cad (+), P120 (+)
DCIS	59	yes	3		neg	neg	equiv (2+)	ND	15%	CD10 (+), P63 (+), Calponin (+)
DCIS	54	yes	3		neg	neg	equiv (2+)	ND	25%	CK5/6 (−), EGFR (±)
DCIS	46	no	3	IIB	neg	neg	equiv (2+)	ND	40%	CK5 (+), EGFR (±), E-cad (+)
DCIS	65	yes			neg	neg	equiv (2+)	ND	45%	
DCIS	32	no		IIA	neg	neg	equiv (2+)	ND		
DCIS	50	no	3	IIA	neg	neg	pos (3+)	pos	15%	CK5/6 (−), EGFR (−)
DCIS	56	yes		I	neg	neg	pos (3+)	pos	20%	EGFR (−), E-cad (+)
DCIS	41	no			neg	neg	pos (3+)	pos		
DCIS	33	no			pos	ND	neg (−)		5%	CK5/6 (−), calponin (−)
DCIS	42	no			pos	pos	neg (−)		10%	CK5/6 (−), EGFR (−)
DCIS	36	no		IIA	pos	pos	neg (−)		10%	E-cad (+), EGFR (−)
DCIS	41	yes			pos	pos	neg (1+)		7%	CK5/6 (−), calponin (+), CD10 (−), E-cad (+), P120 (+)
DCIS	48	no		IIIA	pos	pos	equiv (2+)		3%	CK5/6 (−), EGFR (−)

DCIS	43	no			pos	pos	equiv (2+)		5%	
DCIS	39	no	2		pos	pos	equiv (2+)		5%	CK5/6 (−), EGFR (−), P120 (+), E-cad (+)
DCIS	63	no			pos	pos	equiv (2+)		7%	EGFR (−), E-cad (+)
DCIS	51	no	2		pos	pos	equiv (2+)		7%	EGFR (−), P120 (+), E-cad (+)
DCIS	51	no	2	IIA	pos	pos	equiv (2+)		8%	
DCIS	43	no			pos	pos	equiv (2+)		10%	EGFR (−), CK5/6 (−), P63 (+)
DCIS	46	no	2		pos	pos	equiv (2+)		10%	EGFR (+), P120 (+), E-cad (+), CK5/6 (+)
DCIS	41	no			pos	pos	equiv (2+)		10%	CK5/6 (−), EGFR (−)
DCIS	39	no		IIA	pos	pos	equiv (2+)		30%	CK5/6 (−), P120 (+), E-cad (+)
DCIS	51	yes			pos	pos	equiv (2+)		50%	CK10 (+), calponin (−)
DCIS	70	yes			pos	pos	neg (−)		15%	EGFR (−), P120 (+)
DCIS	67	yes		IIA	pos	pos	neg (1+)		20%	CK5/6 (−), EGFR (−)
Benign - adenosis	42	no								
Benign - adenosis	49	no								
Benign - adenosis	45	no								
Benign - adenosis	38	no								
Benign - adenosis	48	no								
Benign - adenosis	55	yes								
Benign - adenosis	51	no								
Benign - adenosis, intraductal papilloma	44	no								
Benign - adenosis, intraductal papilloma	46	no								
Benign - adenosis, mastitis	42	no								
Benign - adenosis, phyllodes	38	no								
Benign - adenosis, apocrine metaplasia	51	no								
Benign - adenosis, apocrine metaplasia	38	no								
Benign - adenosis, fibroadenoma, apocrine metaplasia	52	no								

Benign - adenosis, fibroadenoma	42	no								
Benign - adenosis, fibroadenoma	24	no								
Benign - adenosis, fibroadenoma	26	no								
Benign - adenosis, fibroadenoma	45	no								
Benign - adenosis, fibroadenoma	35	no								
Benign - adenosis, fibroadenoma	45	no								
Benign - adenosis, fibroadenoma	42	no								
Benign - adenosis, fibroadenoma	46	no								
Benign - adenosis, fibroadenoma	41	no								
Benign - adenosis, fibroadenoma	38	no								
Benign - adenosis, fibroadenoma	40	no								
Benign - adenosis, fibroadenoma	53	no								
Benign - adenosis, fibroadenoma	40	no								
Benign - adenosis, inflammation	33	no								
Benign - adenosis, inflammation	56	yes								
Benign - adenosis, inflammation	33	no								
Benign - adenosis, inflammation	48	no								
Benign - adenosis, inflammation	40	no								
Benign - fibroadenoma	41	no								
Benign - fibroadenoma	38	no								
Benign - fibroadenoma	34	no								
Benign - fibroadenoma	43	no								
Benign - fibroadenoma	41	no								
Benign - fibroadenoma	41	no								
Benign - fibroadenoma	46	no								

Benign - fibroadenoma	62	yes								
Benign - fibroadenoma	37	no								
Benign - fibroadenoma	36	no								
Benign - fibroadenoma, infarction	45	no								
Benign - fibrous epithelial, ductal hyperplasia	42	no								
Benign - fibrous epithelial, ductal hyperplasia	41	no								
Benign - granulomatous inflammation	35	no								
Benign - intraductal papilloma	51	no								
Benign - intraductal papilloma	58	yes								
Benign - lobular hemangioma	48	no								
Benign - lobular hyperplasia	50	no								
Benign - lobular hyperplasia	53	no								
Benign - phyllodes	45	no								
Healthy	46									
Healthy	26									
Healthy	27									
Healthy	44									
Healthy	47									
Healthy	52									
Healthy	40									
Healthy	32									
Healthy	74									
Healthy	62									
Healthy	42									
Healthy	42									
Healthy	38									
Healthy	40									
Healthy	47									
Healthy	50									
Healthy	66									

Healthy	60									
Healthy	70									
Healthy	33									
Healthy	30									
Healthy	29									
Healthy	52									
Healthy	28									
Healthy	48									
Healthy	39									
Healthy	42									
Healthy	60									
Healthy	27									
Healthy	27									
